# Au-Capped GaAs Nanopillar Arrays Fabricated by Metal-Assisted Chemical Etching

**DOI:** 10.1186/s11671-017-2219-1

**Published:** 2017-07-05

**Authors:** Hidetaka Asoh, Ryota Imai, Hideki Hashimoto

**Affiliations:** Department of Applied Chemistry, Kogakuin University, 2665-1 Nakano, Hachioji, Tokyo, 192-0015 Japan

**Keywords:** GaAs, Metal-assisted chemical etching, Au nanodot arrays

## Abstract

GaAs nanopillar arrays were successfully fabricated by metal-assisted chemical etching using Au nanodot arrays. The nanodot arrays were formed on substrates by vacuum deposition through a porous alumina mask with an ordered array of openings. By using an etchant with a high acid concentration and low oxidant concentration at a relatively low temperature, the area surrounding the Au/GaAs interface could be etched selectively. Under the optimum conditions, Au-capped GaAs nanopillar arrays were formed with an ordered periodicity of 100 nm and pillar heights of 50 nm.

## Background

III–V compound semiconductors have attracted attention as next-generation materials and potential alternatives to silicon-based semiconductors because of their excellent properties including superior carrier mobility and direct band gap. Nanostructures with ordered periodicity and/or high aspect ratio are considered to be important element in various applications including optical and optoelectronic devices because of their low cost and high conversion efficiency compared to conventional thin film-based devices [1–4]. In general, to fabricate low-dimensional semiconductors (e.g., nanowires), dry processes such as molecular beam epitaxy, vapor–liquid–solid epitaxy, and metal-organic vapor-phase epitaxy are used [1, 5–7]. Although these methods have many advantages including high patterning accuracy, their drawbacks include high cost and size limitations of the patterning area in practical applications. Therefore, alternative methods that enable the simple and cost-effective fabrication of nanostructures are needed.

Metal-assisted chemical etching, which was proposed by Li and Bohn in 2000 [8], is a commonly used fabrication method owing to its relative simplicity and low cost. Recent studies have demonstrated that metal-assisted chemical etching can be applied to fabricate complex nanostructures such as deep, straight nanopores, helical nanopores, sloping channels, cycloids, and spirals [4, 9–12]. However, since the report by Li et al., most studies have reported the fabrication of silicon nanostructures; few investigations have focused on the nanofabrication of III-V compound semiconductors [13, 14], and the formation of ordered nanometer-scale structures on GaAs substrates is particularly poorly understood. To expand the range of applications of metal-assisted chemical etching, it is desirable to develop a nanofabrication method for III-V compound semiconductors that does not depend on the dimensions of the resultant patterns.

In a previous study, we fabricated microbump arrays of InP [15] and line patterns and pillar arrays of GaAs [16] using metal-assisted chemical etching. However, the dimensions of the resultant patterns (e.g., periodicity and widths of the line patterns) ranged from several micrometers to several tens of micrometers. To the best of our knowledge, no study has reported the formation of ordered GaAs nanostructures with submicron-scale or smaller periodicity using metal-assisted chemical etching for the following reasons: (1) it is difficult to control the shape and size of noble metals used as a catalyst on the nanometer scale and (2) the etching phenomenon of GaAs is less well understood compared to the case of silicon. Thus, we attempted to clarify the etching mechanism of GaAs on the nanometer scale. In this study, we demonstrate that ordered nanopillar arrays with a periodicity of 100 nm can be fabricated on GaAs substrates using metal-assisted chemical etching with a patterned Au catalyst. The effects of etchant composition and etching time on the morphology of the etched GaAs substrate are also investigated.

## Methods

**Fig. 1:**
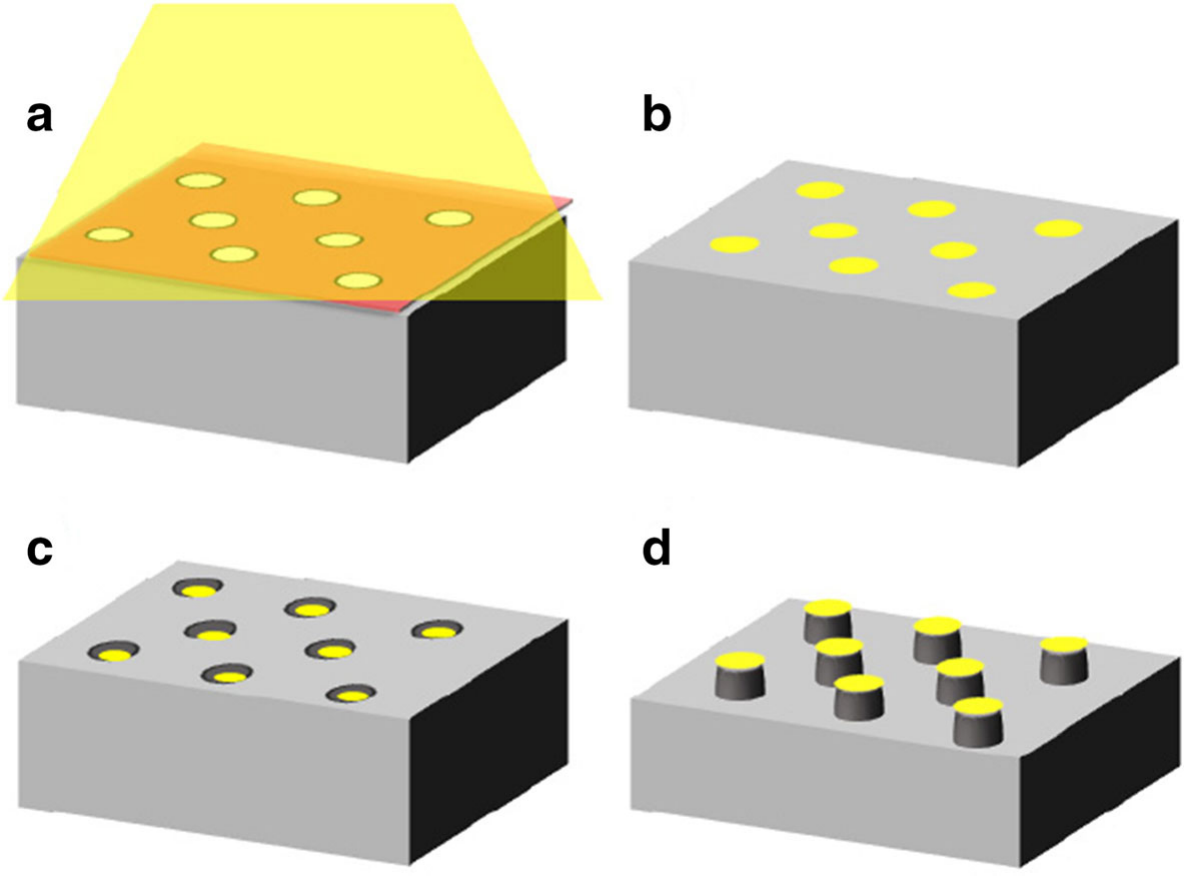
Schematic model of the fabrication of GaAs nanopillar arrays. **a** Vacuum deposition of Au on a GaAs substrate through porous alumina mask. **b** Removal of the mask. **c**, **d** Chemical etching of GaAs using Au nanodot arrays as a catalyst

The principle of the fabrication of GaAs nanopillar arrays via metal-assisted chemical etching is shown schematically in Fig. 1. A through-hole porous alumina mask with an ordered array of openings was prepared by two-step anodization followed by two-layer anodization [17]. The first anodization was carried out on electrochemically polished aluminum (99.99% purity) at a constant voltage of 40 V in 0.3 mol dm^−3^ oxalic acid at 30 °C for 3 h. The anodization voltage of 40 V is well-established as a self-ordering condition that produces a highly ordered pore arrangement in anodic alumina [18].

After the first anodization, the first anodized alumina layer was removed in a mixed solution of phosphoric acid and chromic acid at 80 °C. Subsequently, the second anodization was conducted for 1.5 min under the same conditions as the first anodization. Based on the principle of two-layer anodization, the specimen was anodized again at a constant voltage of 40 V in 12 mol dm^−3^ sulfuric acid at 5 °C for 8 min to prepare a sacrificial alumina layer. The through-hole porous alumina mask was formed by dissolving the sacrificial alumina layer in 2 wt% phosphoric acid at 30 °C for 20 min. Further chemical etching was conducted in 5 wt% phosphoric acid at 30 °C for 15 min to increase the pore diameter of the alumina mask.

After rinsing the alumina mask in distilled water, the obtained alumina mask was set on an n-type GaAs substrate [Si-doped, 2.35–2.67 × 10^−3^ Ω cm, (100) crystal orientation]. Subsequently, a 30-nm-thick Au layer was evaporated through the alumina mask using a vacuum deposition system by the resistance heating formula (ULVAC KIKO Inc., VPC-410) with a pressure below 1 × 10^−3^ Pa (Fig. 1a). The thickness of the Au layer was measured using a quartz crystal microbalance, and the deposition rate of Au was 0.02 nm s^−1^. After metal deposition, the alumina mask was removed in 5 wt% phosphoric acid at 25 °C for 30 min (Fig. 1b).

The Au-coated GaAs substrate was chemically etched in HF containing KMnO_4_ (Fig. 1c). KMnO_4_ acts as an oxidizing agent in an acidic solution [19–22]. The morphologies of the alumina mask, deposited Au layer, and etched GaAs substrate were evaluated by field-emission scanning electron microscopy (FE-SEM; JEOL JSM-6701F). The chemical composition of etched GaAs substrate was evaluated by auger electron spectroscopy (AES; JEOL JAMP-9500F). Auger electron spectra are easily acquired from selected points or areas of the surface. Here, AES elemental mapping image was acquired with an accelerating voltage and emission current of 30 kV and 15 nA, respectively.

## Results and Discussion

In metal-assisted chemical etching, it is essential to precisely control the dimensions of the metal catalyst to obtain the desired design on the substrate surface. Because the morphology of the resultant structure depends on the initial geometric pattern and dimensions of the metal catalyst, a patterned metal catalyst is required to fabricate ordered nanostructures on semiconductor surfaces. In this study, an alumina mask with an ordered array of openings was used to control the size and arrangement of the metal catalyst. For a dry metal-deposition process, the thickness of the mask is critical because metal deposition through a thick mask with narrow apertures is physically difficult. In the case of porous alumina, the thickness of the mask can be adjusted with high reproducibility by changing the anodization time. Here, an approximately 300-nm-thick through-hole porous alumina mask was prepared on a GaAs substrate. The alumina mask was set with its surface facing upward. The top and bottom diameters of the openings in the alumina mask were approximately 80 and 70 nm, respectively. The slightly larger diameter of the top opening compared to the bottom opening was attributed to chemical etching during the preparation of the alumina mask.

**Fig. 2:**
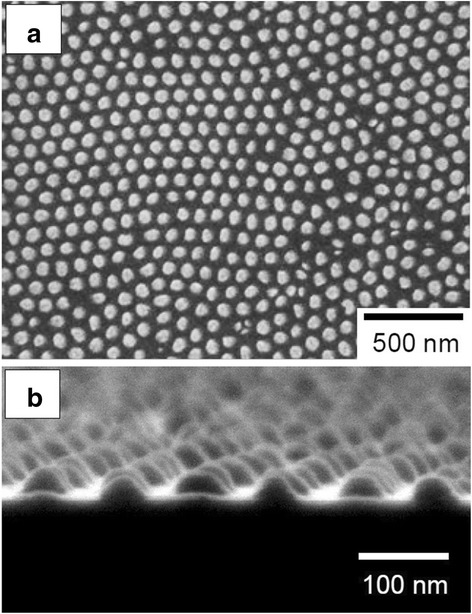
**a** Surface and **b** cross-sectional SEM images of an Au nanodot array formed on a GaAs substrate through an anodic porous alumina mask

Figure 2 shows a typical well-ordered Au nanodot array on a GaAs substrate. The nanodot array corresponds to the configuration of the self-ordered pore array in the anodic alumina mask, as shown in Fig. 2a. Although the controllability of Au deposition should be further improved, the metal deposition through the alumina mask demonstrated herein is suitable for the large-scale production of ordered noble-metal dot patterns on semiconductor substrates in terms of the simplicity and efficiency of the fabrication process. Note that each Au nanodot had nearly the same diameter of approximately 70 nm; this diameter was determined by the pore size of the bottom part of the alumina mask, while the heights of Au nanodots were primarily determined by the deposition time. In this study, the height of each Au nanodot was adjusted to be ~30 nm, as show in Fig. 2b.

After the formation of Au dot arrays on GaAs substrates, the specimens were immersed in a solution of HF and KMnO_4_ for metal-assisted chemical etching. In conventional metal-assisted chemical etching, the etching proceeds locally at the interface between the catalyst and the underlying substrate, resulting in the formation of pores or trenches in the direction perpendicular to the substrate, and the metal catalyst sinks into the semiconductor as shown in Fig. 1c. The use of an etchant composed of a high acid concentration and a low oxidant concentration is considered to promote smooth consumption of the generated positive holes (h^+^) at the metal/semiconductor interface. In this study, the oxidation of GaAs at the Au/GaAs interface is expected to proceed directly by the generated h^+^.

**Fig. 3:**
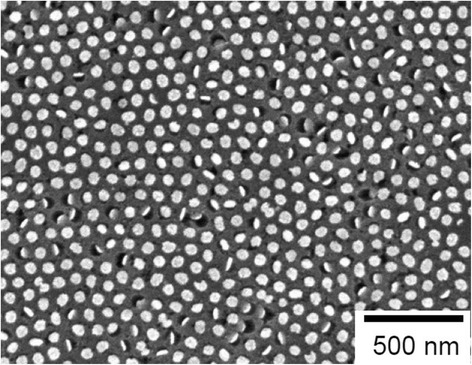
SEM image of top surface of GaAs substrate after Au-assisted chemical etching in solution containing 0.001 mol dm^−3^ KMnO_4_ and 20 mol dm^−3^ HF for 600 s at 45 °C

Figure 3 shows a typical SEM image of the etched GaAs surface using the patterned Au catalyst. Chemical etching was conducted in solution containing 0.001 mol dm^−3^ KMnO_4_ and 20 mol dm^−3^ HF at a relatively high temperature of 45 °C. In this study, the concentration of KMnO_4_ was low (0.001 mol dm^−3^) to suppress lateral etching. According to previous reports by DeJarld et al. and Cheung et al., the lateral etching rate increased with increasing oxidizing agent (KMnO_4_) concentration [19, 21].

Au catalysts, which were detected as circular bright spots, were observed inside pores in many areas of the GaAs substrate, as shown in Fig. 3. The diameter of the pores observed in Fig. 3 coincided with the sizes of the deposited Au nanodots shown in Fig. 2. These results indicate that conventional metal-assisted chemical etching, which is shown schematically in Fig. 1c, occurred only at the Au/GaAs interface and proceeded anisotropically perpendicular to the substrate, i.e., in the <100> direction.

**Fig. 4:**
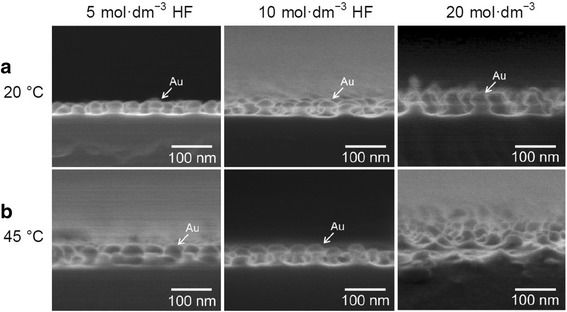
SEM images of GaAs nanopillar arrays fabricated by Au-assisted chemical etching in solutions containing 0.01 mol dm^−3^ KMnO_4_ and 5, 10, or 20 mol dm^−3^ HF for 5 s at **a** 20 and **b** 45 °C

In metal-assisted chemical etching, the etchant composition and etching temperature affect the dynamics of carrier diffusion, oxidation, and product removal [19]. To open up new applications of etched GaAs substrate, we attempted to fabricate GaAs nanopillar arrays by changing the conditions of metal-assisted chemical etching. Figure 4 shows typical cross-sectional SEM images of etched GaAs surfaces obtained using the patterned Au catalyst. By increasing the concentration of KMnO_4_, the morphology of the resultant structure could be changed. In all cases, GaAs nanopillars arranged hexagonally over the entire specimen area were obtained. The tips of the pillars were slightly tapered as a result of lateral etching. The periodicities of the GaAs nanopillar arrays were approximately 100 nm, corresponding to those of the Au dot arrays used as catalyst and the pores of the porous alumina used as the initial mask. To our knowledge, the dimensions (e.g., periodicity) of the structures obtained on GaAs via metal-assisted chemical etching in this study are smaller than those reported for other GaAs structures [19–22].

When etching was conducted at a relatively low temperature of 20 °C, Au catalyst was observed at the tip of each pillar, as indicated by arrows. Figure 4a shows that the etching rate increased with increasing HF concentration at the same oxidant concentration. At the high HF concentration of 20 mol dm^−3^, the residual GaAs pillar height was the highest.

Contrary to expectation that the oxidation of GaAs at the Au/GaAs interface proceeds by the generated h^+^, no chemical dissolution in the area of contact between the Au catalyst and the underlying GaAs substrate was observed in the case of Fig. 4. The etching pattern is considered to be dependent on the temperature of the etchant. At low temperature (e.g., 20 °C), the rate of h^+^ consumption at the Au/GaAs interface is thought to be lower than the rate of h^+^ injection; thus, h^+^ diffused into the area surrounding Au-coated GaAs. Eventually, a GaAs nanopillar was formed under the area of contact between the Au catalyst and the underlying GaAs substrate because site-selective etching occurred on the exposed GaAs surface. In other words, the Au nanodots also acted as a protective mask to prevent the dissolution of the GaAs substrate. This etching phenomenon as shown schematically in Fig. 1d is called inverse metal-assisted chemical etching [19, 22]. In 2010, we also demonstrated the formation of InP microbump arrays using inverse metal-assisted chemical etching under UV irradiation [15]. In contrast to conventional metal-assisted chemical etching, inverse metal-assisted chemical etching proceeds in exposed III–V compound semiconductor’s surfaces around metal-coated areas by the diffusion of h^+^ from metal catalyst and subsequent site-selective chemical etching. Such a unique etching behavior has not been observed in silicon materials.

When metal-assisted chemical etching is conducted at a high temperature of 45 °C, the generated h^+^ is expected to be consumed as soon as it reaches the boundary between Au, GaAs, and etchant, resulting in the promotion of vertical etching. Even in this case, however, inverse metal-assisted chemical etching occurred. As shown in Fig. 4b, the etching rate increased with increasing HF concentration in the same way as etching behavior of Fig. 4a. However, at a high etching temperature of 45 °C and a high HF concentration of 20 mol dm^−3^, the Au catalysts were detached from the tips of the GaAs pillars because the excess h^+^ generated by the relatively high temperature, even in the short etching time of 5 s, promoted the lateral etching of GaAs in the presence of the Au catalyst. The shape controllability of the pillars will be improved by the additional optimization of etching conditions (e.g., etchant composition, concentration, and temperature). Attempts to clarify the effects of the concentration of oxidizing agent on the generation of h^+^ and morphology of etched GaAs are currently underway.

**Fig. 5:**
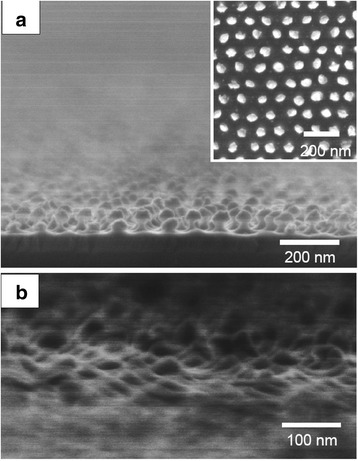
Cross-sectional SEM images of a GaAs nanopillar array fabricated by Au-assisted chemical etching at 20 °C for **a** 10 and **b** 60 s in a solution containing 20 mol dm^−3^ HF and 0.01 mol dm^−3^ KMnO_4_. *Inset* shows a surface image of an Au-capped GaAs nanopillar array

**Fig. 6:**
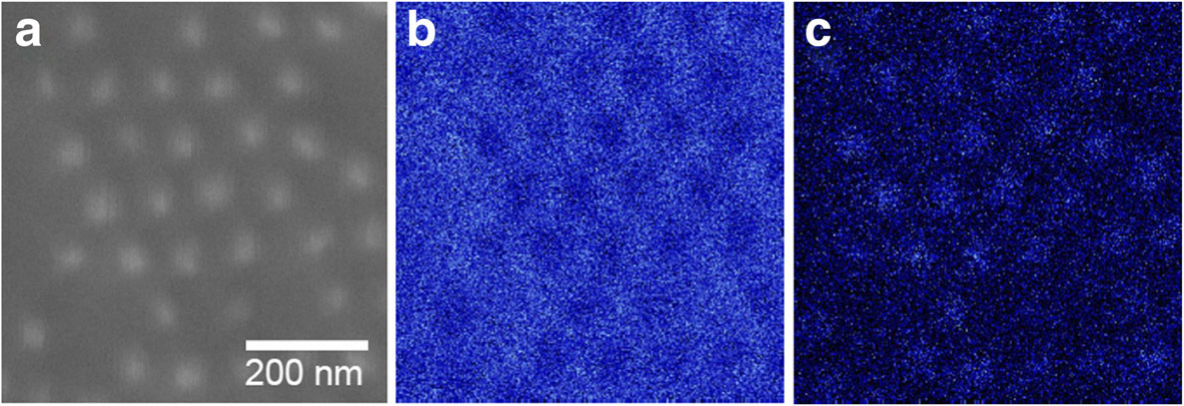
**a** Image of GaAs substrate after Au-assisted chemical etching and corresponding AES maps for **b** Ga and **c** Au. The etching conditions were the same as those in Fig. 5a

To examine the effect of etching time on the geometry of the etched GaAs structure, chemical etching was prolonged in 20 mol dm^−3^ HF and 0.01 mol dm^−3^ KMnO_4_ at a relatively low temperature of 20 °C. As shown in the cross-sectional image in Fig. 5a, the depth of the GaAs nanopillars reached ~50 nm. One of the notable features of the GaAs nanopillar arrays obtained in this study is that the tip of each pillar was covered with Au, as shown in the inset of Fig. 5a. Figure 6 shows the AES elemental analysis of the same specimen. The AES maps for Ga and Au of the etched GaAs indicate the presence of Au at the tip of each pillar even after metal-assisted chemical etching for 10 s.

Because the pillar height was determined primarily by etching time, etching was further prolonged from 10 s to 1 min to form arrays of higher pillars on GaAs. However, the prolonged etching time of 1 min resulted in decreased pillar height, as shown in Fig. 5b. The decrease in pillar height was attributed to lateral etching in the presence of Au catalyst and the subsequent detachment of the Au dots used as catalyst.

Although metal-assisted chemical etching, which precisely controls the diffusion of h^+^ from metal catalyst, has not yet been fully accomplished, the nanofabrication of III-V compound semiconductors using metal-assisted chemical etching offers a promising alternative for the design of ordered three-dimensional structures without the use of dry processes. In addition, the obtained Au-capped GaAs nanopillar arrays have potential technological and scientific applications in optoelectronic devices such as solar cells that employ plasmonic nanostructures to enhance light trapping [23, 24].

## Conclusions

In summary, we have demonstrated the fabrication of ordered GaAs nanopillar arrays on GaAs (100) substrates via Au-assisted chemical etching. Au nanodot arrays with hexagonal lattice patterns and ordered periodicities of 100 nm were formed by vacuum deposition through a porous alumina mask. The Au nanodots had diameters of approximately 70 nm, corresponding to the diameter of the bottom part of the alumina mask, and served as a catalyst and a protective mask. At relatively low temperature, Au-capped GaAs nanopillar arrays could be formed by site-selective etching in the surrounding exposed GaAs surface. These findings provide the first evidence for the more precise control of nanostructures on GaAs substrates using a feasible approach based on metal-assisted chemical etching. The unconventional lithography technique for the nanofabrication of III–V compound semiconductors presented in this communication overcomes the drawbacks of conventional methods and has potential technological and scientific applications in various research fields.

## Abbreviations

AES: Auger electron spectroscopy
FE-SEM: Field-emission scanning electron microscopy
